# A Novel Laser-Doppler Flowmetry Assisted Murine Model of Acute Hindlimb Ischemia-Reperfusion for Free Flap Research

**DOI:** 10.1371/journal.pone.0066498

**Published:** 2013-06-20

**Authors:** Tolga Taha Sönmez, Othman Al-Sawaf, Gerald Brandacher, Isabella Kanzler, Nancy Tuchscheerer, Mersedeh Tohidnezhad, Anastasios Kanatas, Matthias Knobe, Athanassios Fragoulis, René Tolba, David Mitchell, Thomas Pufe, Christoph Jan Wruck, Frank Hölzle, Elisa Anamaria Liehn

**Affiliations:** 1 Department of Oral and Maxillofacial Surgery, Medical Faculty, RWTH Aachen University, Aachen, Germany; 2 Institute of Anatomy and Cell Biology, Medical Faculty, RWTH Aachen University, Aachen, Germany; 3 Institute for Molecular Cardiovascular Research (IMCAR), Medical Faculty, RWTH Aachen University, Aachen, Germany; 4 Department of Plastic and Reconstructive Surgery, Johns Hopkins University School of Medicine, Baltimore, Maryland, United States of America; 5 Department of Thoracic and Cardiovascular Surgery, Johann Wolfgang Goethe University, Frankfurt/Main, Germany; 6 Department of Oral and Maxillofacial Surgery, Leeds Teaching Hospitals and St James Institute of Oncology, Leeds Dental Institute, Leeds, United Kingdom; 7 Department of Orthopaedic and Trauma Surgery, Medical Faculty, RWTH Aachen University, Aachen, Germany; 8 Institute of Laboratory Animal Science and Experimental Surgery, Medical Faculty, RWTH Aachen University, Aachen, Germany; 9 Department for Oral and Maxillofacial Surgery, Mid-Yorkshire and Leeds Teaching Hospitals, The Mid Yorkshire Hospitals NHS Trust, Wakefield, United Kingdom; University of Louisville, United States of America

## Abstract

Suitable and reproducible experimental models of translational research in reconstructive surgery that allow *in-vivo* investigation of diverse molecular and cellular mechanisms are still limited. To this end we created a novel murine model of acute hindlimb ischemia-reperfusion to mimic a microsurgical free flap procedure. Thirty-six C57BL6 mice (n = 6/group) were assigned to one control and five experimental groups (subject to 6, 12, 96, 120 hours and 14 days of reperfusion, respectively) following 4 hours of complete hindlimb ischemia. Ischemia and reperfusion were monitored using Laser-Doppler Flowmetry. Hindlimb tissue components (skin and muscle) were investigated using histopathology, quantitative immunohistochemistry and immunofluorescence. Despite massive initial tissue damage induced by ischemia-reperfusion injury, the structure of the skin component was restored after 96 hours. During the same time, muscle cells were replaced by young myotubes. In addition, initial neuromuscular dysfunction, edema and swelling resolved by day 4. After two weeks, no functional or neuromuscular deficits were detectable. Furthermore, upregulation of VEGF and tissue infiltration with CD34-positive stem cells led to new capillary formation, which peaked with significantly higher values after two weeks. These data indicate that our model is suitable to investigate cellular and molecular tissue alterations from ischemia-reperfusion such as occur during free flap procedures.

## Introduction

Microsurgical composite tissue transfer has become the gold standard in reconstructive surgery [1], [2]. Even though ische mia-reperfusion injury (IRI) is inevitable in free flap surgery and composite tissue allotransplants, and may be expected to incur total or partial flap necrosis or complicated re-exploration surgery [2], its precise biochemical and cellular mechanisms are still not completely understood.

It is known that reperfusion injury causes most early tissue morbidity and mortality [3], [4]. IRI and post-ischemic reperfusion lead to acute vascular leakage and to nerve and particularly skeletal muscle damage [3], [5]. Post-ischemic reperfusion also causes a burst in reactive-oxygen-species (ROS), alterations in mitochondrial function, inflammation, and ultimately, tissue necrosis [6]–[8].

Several models of acute hindlimb ischemia are currently in use for study of the effects of IRI and of potential therapeutic interventions [9], [10]. However, it still remains a challenge to replicate the best research conditions in murine hindlimb models, which can mimic the perioperative situation of microsurgical composite tissue transfer. Sufficient occlusion of all branches of the femoral and iliac vessels, including the rich collateral blood supply from iliac and tail branches of the hindlimb, is essential in order to induce complete, acute ischemia. Even as sufficient occlusion of hindlimb vessels is assured, however, care must also be taken to avoid the irreversible tissue damage and neuromuscular deficits which an uncontrolled crushing force can cause.

Previously described techniques seem less suitable for analysis of cellular and molecular alterations within skin and muscle, as they entail ischemia-reperfusion, which may cause irreversible damage. Usually, this does not occur in free flap procedures done in a clinical setting.

To address this issue, we designed a Laser-Doppler Flowmetry assisted murine hindlimb model to create complete acute ischemia-reperfusion that is reproducible and suitable for translational research related to composite tissue transplantation and free flap procedures.

## Materials and Methods

### Animal Protocol

All experiments were conducted in accordance with German legislation governing animal studies. The Principles of Laboratory Animal Care were followed. The animal experiments were approved by the “Landesamt für Natur, Umwelt und Verbraucherschutz Nordrhein-Westfalen” (Reg. 8.87–50.10.37.09.209).

Adult C57BL6 mice (10–12 weeks, 25–28 g) were housed in filter-top cages under SPF-conditions according to the FELASA guidelines at 22+/−1°C, 50+/−20% relative humidity and a 12 h light/dark cycle. Water and standard rodent diet (Sniff, Soest, Germany) was given ad libitum. Animals were allowed to acclimatize to their surroundings for at least one week before procedures were performed.

**Figure 1:**
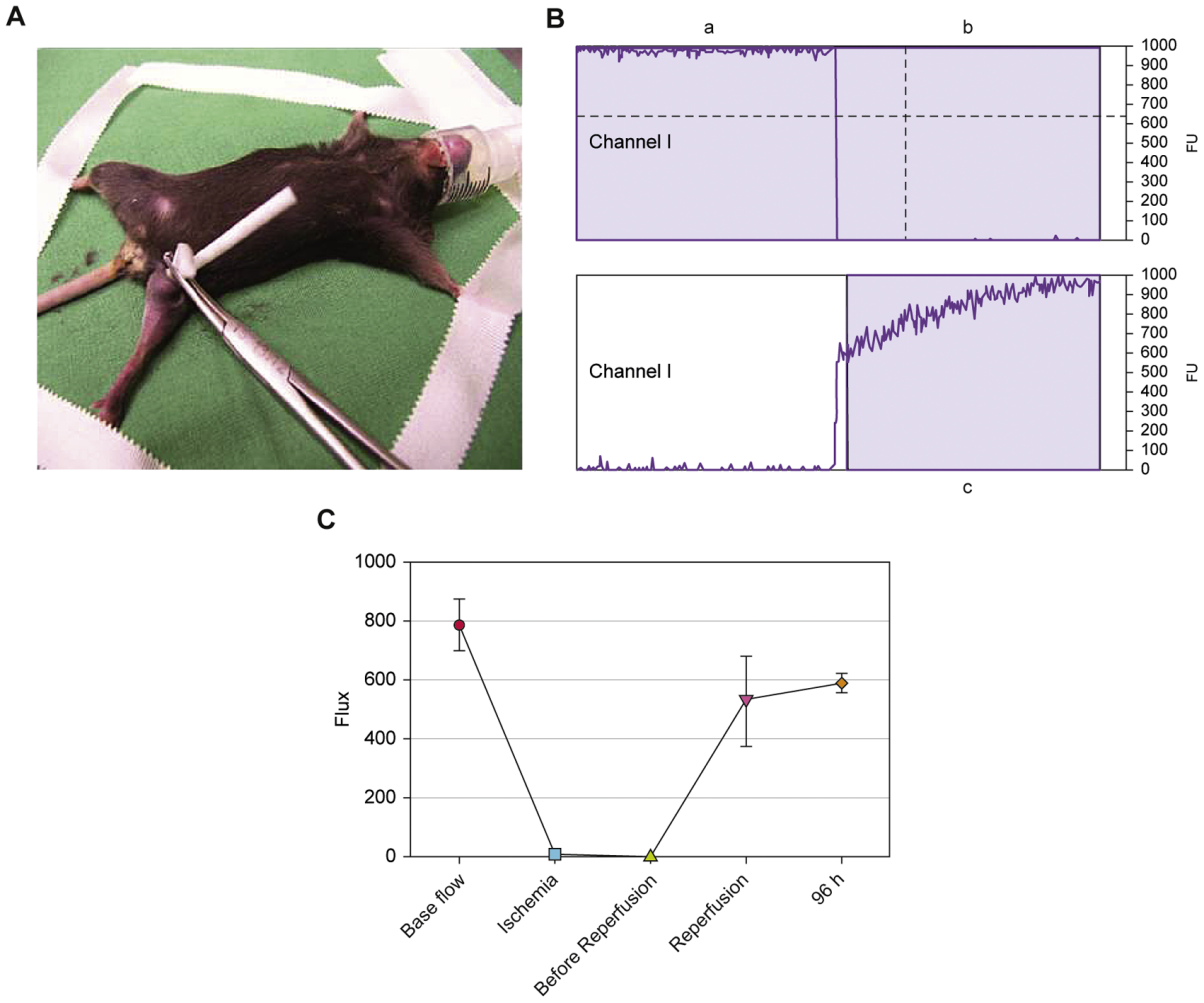
Murine model of Laser-Doppler Flowmetry assisted acute ischemia-reperfusion in hindlimb. (A) Anaesthetized mouse with ligated hindlimb. The vessel loop was fixed advancing a small, solid plastic tube with a mosquito clamp throughout the whole ischemia period. (B–C) Blood perfusion to the hindlimb was assessed using Laser-Doppler Flowmetry (moorVMS-LDF1) (a) before ischemia, (b) during ischemia and (c) directly when reperfusion is induced. Error bars represent Standard Error of the Mean (SEM).

A total of thirty-six mice (n = 6) were assigned randomly into 6 groups: Control, 6 h, 12 h, 96 h, 120 h and 14d of reperfusion. For the experiments, the mice were anaesthetized (1,5–2 Vol% isoflurane) and placed on a heating pad to maintain their body temperature at a constant 37°C throughout the experiment. One randomly assigned hindlimb (3 right side, 3 left side) was subjected to 4 hours of ischemia by occlusion of overall blood perfusion by means of a vessel loop (Maxi-white size-2 VesseloopsTM MEDICA Europe, Oss, the Netherlands) as tourniquet and clamp (Fig. 1A). The contralateral hindlimb was not subjected to ischemia. The vessel loop was placed precisely above the greater trochanter and fixed by the advancement of a small, solid plastic tube with a mosquito clamp. To prevent dehydration and sustain microcirculation, 1 ml per hour of 0.9% NaCl was given subcutaneously. After the period of ischemia, the vessel loop was removed to allow reperfusion. Mice were euthanized by cervical dislocation at 6 h, 12 h, 96 h, 120 h or 14 days after reperfusion, respectively for each group. Tissue samples were subsequently harvested and prepared for immunofluorescence and for histomorphological and immunohistochemical analysis.

Additionally, the walking behavior of the mice was monitored daily using a self-defined score for neuromuscular recovery.

### Blood Flow Determination

In order to ensure successful, complete occlusion of overall blood perfusion to the hindlimb, and to assess the tissue microcirculation after reperfusion, a Laser-Doppler Flowmeter (moorVMS-LDF1, Moor-Instruments Ltd, Devon UK) was used. The optic probe was positioned at a right angle above the medial side of the ligated limb and was fixed there. Measurements were taken before occlusion, after occlusion, before reperfusion and immediately once ischemia had terminated. (Fig. 1 B–C). The data were analyzed using moorVMS-PC-Software.

### Quantitative Immunohistochemistry and Immunofluorescence

The skin and muscle of the thigh were primed by removal of the adductor group in one piece, including associated skin and subcutaneous tissue. Tissue samples were embedded in paraffin, and 5 µm serial sections (5–6 per mouse) were stained with haematoxylin and eosin (H&E) for morphological analysis and analysis of inflammatory cell infiltration.

Different specific markers were used to quantify angiogenesis and arteriogenesis at the various endothelial stages. Angiogenic stem cells were stained with anti-CD34 antibody (Abcam) followed by a FITC-conjugated secondary antibody. The content of mature vessels in skin and muscle was analyzed using anti-CD31 antibody (Santa Cruz Biotech.) followed by Cy3-conjugated secondary antibody. Arterioles were marked after double staining by CD31 and SMA (smooth muscle cells, DAKO) followed by a FITC-conjugated secondary antibody. Cytokines were analyzed using anti-VEGF antibody (Santa Cruz Biotech.), followed by a FITC-conjugated secondary antibody and DAPI (Vector Laboratories Inc., USA) used for counterstaining.

Stained cells or vessels were counted in six different fields per section and expressed as cells or vessels/field. For analysis of the size of the arterioles, the diameter of the vessels was measured, with subdivision into small vessels (0 to 20 µm) and medium vessels (20 to 50 µm).

Images were captured using light microscopy (DM 2500, Leica) or fluorescence microscopy (BX50, Olympus) and evaluated with Diskus Software (Hilgers).

### Statistical Analysis

Results were compared using one-way ANOVA, followed by the Newman-Keuls post-hoc test. Differences were considered significant at p<0.05. Data represent the mean (SEM). Statistical analysis was performed using Prism 5 software (GraphPad, San Diego, CA).

## Results

### Assessment of Tissue Regeneration

**Figure 2:**
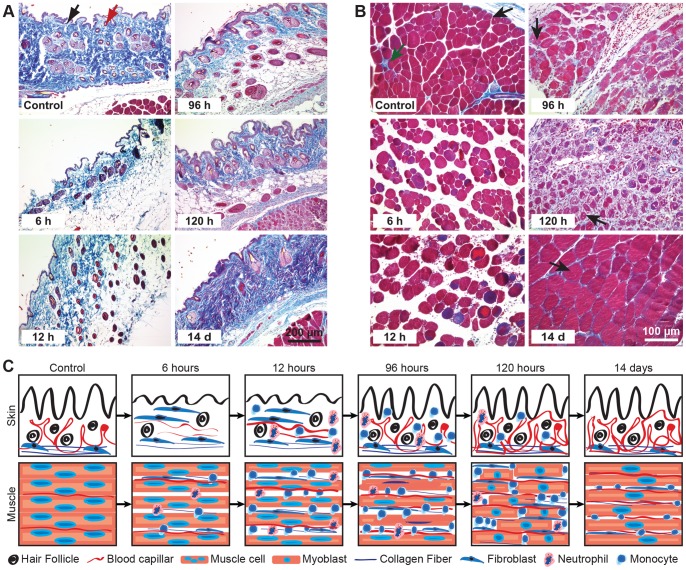
Tissue regeneration after acute ischemia and reperfusion. Haematoxylin and eosin staining showed regeneration of (A) the skin and (B) muscle tissue after 4 hours of ischemia and at different times after reperfusion. Scale bar 200 µm.

The H&E staining revealed massive damage after 4 hours of ischemia and 6 hours of reperfusion. Skin had disintegrated and lost its normal structure, and dermal folds had diminished. However, after 12 hours of reperfusion skin regeneration had begun, as had recruitment and infiltration of inflammatory cells. After 96 hours the skin structure was restored, but the inflammatory cells persisted until 120 hours. After 14 days, skin regeneration was complete, and inflammatory cell infiltration had fallen to base level (Fig. 2A).

In muscle, ischemia-reperfusion had induced swelling of cells and surrounding local edema which persisted until 96 hours (Fig. 2B). Affected muscle cells had lost their myofibril structure after 96 hours and been replaced by young and immature myotubes characterized by central nuclei. By day 14, most nuclei had become peripheral, forming characteristic syncytia. As a consequence, dead muscle cells were progressively replaced by fibrotic tissue (Fig. 2B). After maximum development at 96 hours, extracellular fibrotic tissue was gradually degraded by inflammatory cells and had been replaced after 14 days by regenerated muscle cells.

Inflammatory cells were recruited to the muscle component at a significantly higher level than to the skin. Cells appeared after 6 hours and had infiltrated the affected tissue by 96 and 120 hours. After 14 days, inflammatory cells were still present and were probably contributing to extracellular fibrotic tissue degradation (Fig. 2B).

Following IRI the hindlimb was edematous and remained in abduction, and the mice tried to avoid letting it come into contact with the ground. Neuromuscular deficits decreased until the fourth day, and after two weeks the mice had spontaneously recovered.

### Angiogenesis and Arteriogenesis during Regeneration

**Figure 3:**
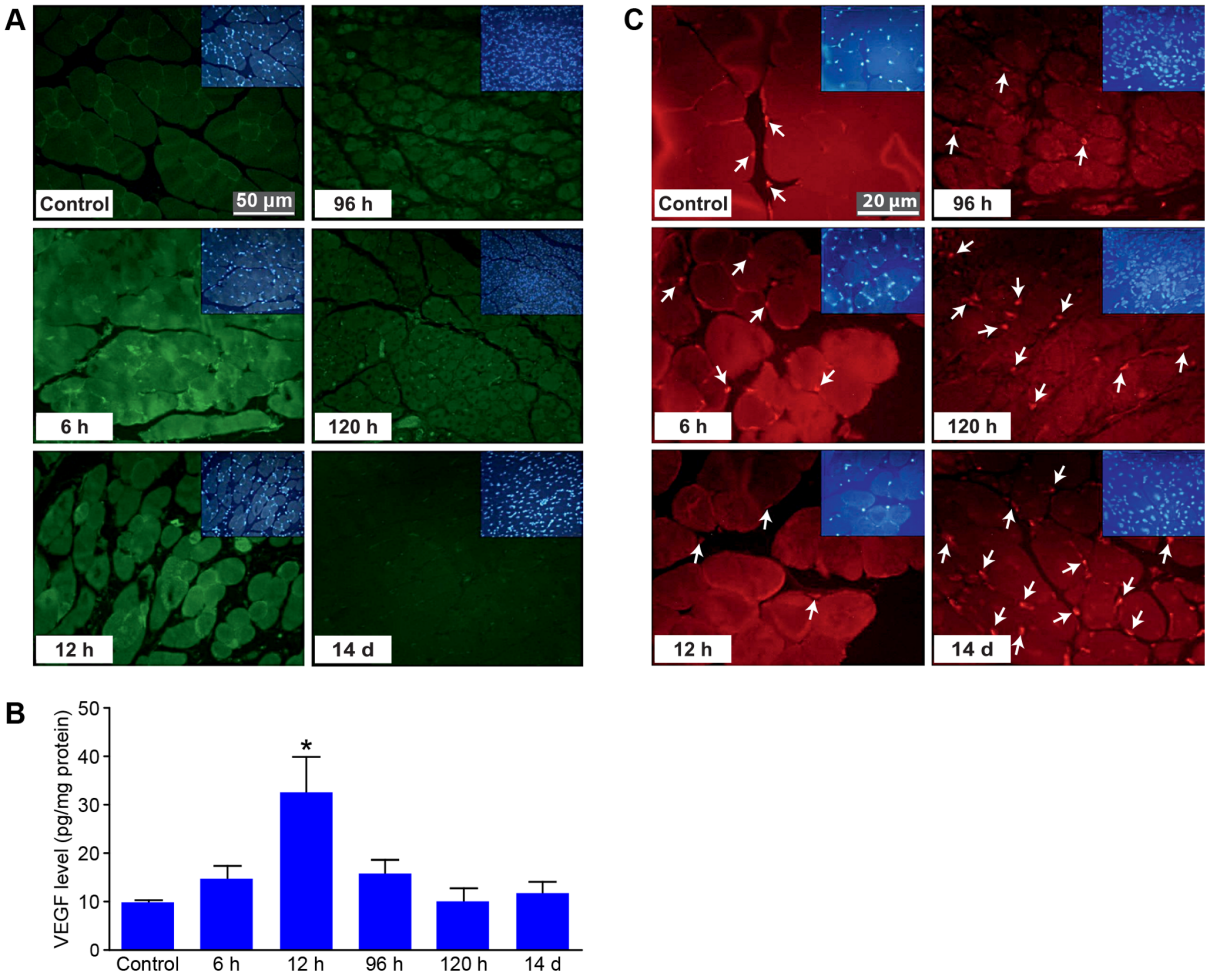
Analysis of angiogenic factors during tissue regeneration. (A) In muscle, VEGF (green) is progressively up-regulated at 6 and 12 hours of reperfusion; 14 days after injury it had returned to base level. Scale bar 50 µm. (B) CD34-positive angiogenic stem cells (red) followed VEGF up-regulation and progressively infiltrated the affected areas starting after 96 hours of reperfusion. Scale bar 20 µm.

To assess angiogenesis in the affected tissue, the main angiogenic cytokine VEGF was analyzed in skin and muscle samples. VEGF in skin was observed predominantly in inflammatory cells (data not shown). In muscle, VEGF was first expressed in inflammatory cells and then progressively upregulated in injured muscle cells at 6 and 12 hours after reperfusion. VEGF had returned to base level by 14 days after reperfusion (Fig. 3A). VEGF upregulation preceded infiltration by angiogenic stem cells stained with CD34. These cells appeared in the 96-hour group and were being recruited progressively at later time points (Fig. 3B).

**Figure 4:**
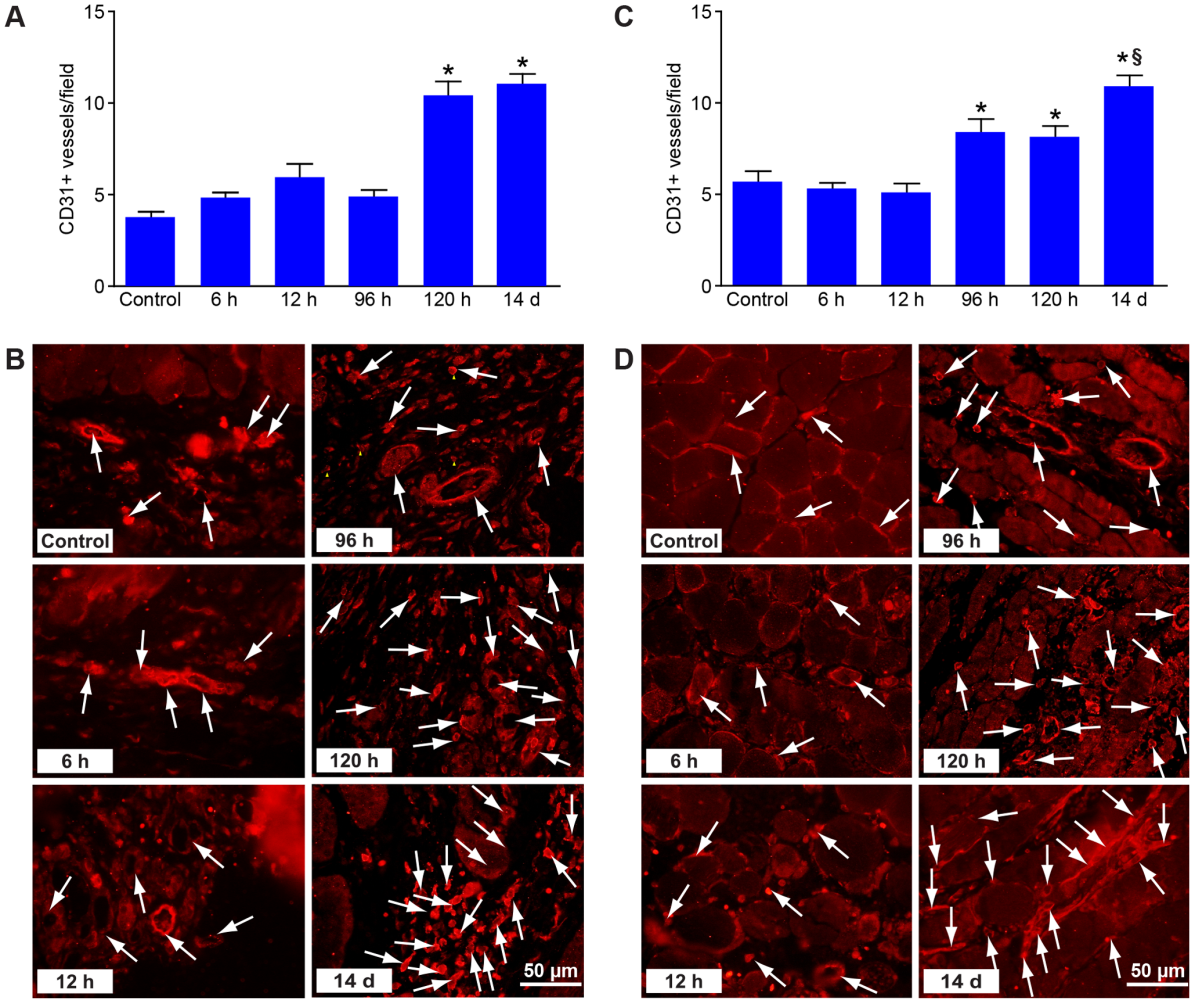
Analysis of angiogenesis during tissue regeneration. (A) Assessment of CD31-positive capillaries at different intervals of reperfusion after 4 h acute ischemia in skin (B) and muscle. *p<0.05. The mean values of CD31 positive capillaries are given in Table 1 and 2, respectively.

Assessment of CD31-positive capillaries revealed that vessel formation was taking place, probably after angiogenic stem cells had matured. In skin, new capillary formation had doubled after 120 hours compared to controls (Fig. 4A, Table 1). In muscle, capillaries appeared earlier, at 96 hours after reperfusion, and had increased at each observation through day 14 (Fig. 4B, Table 2).

**Table 1:** Assessment of CD31-positive capillaries in the skin (n = 6).

	Control	6 h	12 h	96 h	120 h	14 days
**Mean**	3.77	4.80	5.96	4.84	9.78	10.12
**Std. Deviation**	1.4	2.3	3.6	2.9	3.3	3.0
**Std. Error**	0.29	0.32	0.71	0.41	0.63	0.46

**Table 2:** Assessment of CD31-positive capillaries in the muscle (n = 6).

	Control	6 h	12 h	96 h	120 h	14 days
**Mean**	5.63	5.28	5.07	8.35	8.10	10.90
**Std. Deviation**	2.8	2.5	3.2	5.6	3.7	5.0
**Std. Error**	0.64	0.34	0.52	0.76	0.61	0.60

We assessed quantitatively small (up to 20 µm) and mid-sized (up to 50 µm) arterioles after double staining with CD31 and SMA in order to study arteriogenesis. After ischemia, the number of arterioles decreased dramatically. However, they subsequently started to regenerate and had reached control level in the skin after 96 hours.

## Discussion

A suitable experimental model is needed to support translational research in relation to microsurgical composite flap transfer, and to investigate IRI and reperfusion effects. Therefore, we designed a simple, novel model of acute murine hindlimb ischemia-reperfusion using vessel loops.

As previously described, IRI and post-ischemic reperfusion can lead to local as well as remote tissue damage [3], [11]–[14]. Furthermore it has been shown that reperfusion of acutely ischemic limbs may cause remote organ damage e.g. hepatic or renal dysfunction, or muscle or pulmonary damage [3], [11]. Some groups [3], [12] have applied rubber band tourniquets to the hindlimbs bilaterally, as originally described by Rosenthal [15], and have found irreversible tissue damage, resulting in high mortality rates, to be a systemic effect of remote IRI in animals. Most such groups did not use blood flow monitoring to protect the hindlimb from excessive occlusion. Several studies applied rubber bands (Latex O-ring) using the McGivney Hemorrhoidal Ligator (MHL), usually for 2 hours of acute hindlimb ischemia [13], [16], [17]. MHL is used routinely in the clinic for hemorrhoid treatment. Because of the rigidity of rubber bands and their high crushing force with intensity difficult to control, irreversible tissue and neuromuscular damage may result. For this reason, Crawford et al. [9] suggest orthodontic rubber bands using MHL. A drawback of this method, however, lies in finding orthodontic rubber bands of a size and strength to match varying hindlimb girths in mice of different ages (i.e. 8–15 weeks old) or gender to achieve the same ischemia level on all mice. As expected, the investigators found significantly less severe neuromuscular deficits with orthodontic rubber bands on the hindlimb than with the classic MHL method. Another model of acute hindlimb ischemia was presented by Küntscher et al. [18], who used a surgical finger tourniquet to investigate ischemic preconditioning effects. This model did not become established because it used a wide, thin band poorly suited to mice. Nevertheless, Bonheur et al. [10] designed a similar model using a rubber band for measuring tourniquet tension. They were thereby able to induce ischemia for up to 6 hours, whereas only 2 hours of ischemia had been possible in the MHL models. Though it applies graded tension, this model has been criticized for its unwieldiness (involving winches, strain gauges, and pulleys) and its unsuitability especially for molecular imaging [9].

Using tourniquet models of acute ischemia-reperfusion the complete hindlimb blood supply must be occluded for the entire duration of ischemia. This is not the case with femoral artery ligation models of vascular research [19], [20]. Indeed, only a sufficient, non-invasive tourniquet allows complete ischemia of skin, muscle and bone in the hindlimb, with occlusion of collateral blood supply to the posterior thigh from the iliac and tail branches to imitate combined free tissue transfer. A simple, reproducible murine model of hindlimb ischemia-reperfusion, that does not cause irreversible damage, has been missing. In our acute ischemia-reperfusion model, the level of ischemia can easily be adjusted manually using vessel loops. This is indispensable in accounting for individual proportions of animals’ hindlimbs, which vary according to gender, size, age and weight. Using Laser-Doppler Flowmetry we were able to ensure the same sufficient level of ischemia-reperfusion and could reliably adjust the blood occlusion level to hindlimbs of varying circumference.

Regarding animal welfare, other investigators have reported mortality rates up to 70% in mice, despite relatively short ischemia times of two or three hours after release of tourniquet [3], [12], [15]. A distinctly lower mortality rate was observed in our model. It is well known that tourniquet use results in nerve injury and neuromuscular dysfunction in the hindlimb from compression injury to the motor nerve combined with ischemic injury [9], [21]. In our model, reversible neuromuscular deficits in the hindlimb were evident in our observations of rapid recovery in walking behavior.

With regard to angiogenic recovery, we initially observed dramatic damage to skin and muscle tissue following 4 hours of complete ischemia-reperfusion. Despite extensive cellular damage, skin regeneration had begun as early as 12 hours. Myotube formation in muscle was evident after 96 hours. Muscle regeneration progressed for up to two weeks, by which time skin regeneration was complete. In contrast to our findings, Vignaud et al. [14] showed that 3 hours of ischemia-reperfusion induced by rubber bands in mice resulted in muscle fiber death with no apparent survival of muscle fibers, as well as insufficient muscle regeneration by day 14. The contrast between those results and ours likely results from the different experimental hindlimb ischemia method employed.

Furthermore, we found that VEGF upregulation precedes infiltration by CD34-positive angiogenic stem cells, which had appeared by 96 hours of reperfusion and were recruited continuously through day 14 (Fig. 3B). Assessment of CD31-positive capillaries showed that vessel formation did not occur until after 96 hours, probably subsequent to maturation of angiogenic stem cells. We observed an angiogenesis pattern in the muscle component earlier than in the skin. The quantitative analysis of new capillary formation showed a progressive increase through the 14th day. Skin arterioles had reached control level by 96 hours. The final formation of arterioles in muscle was significantly higher than for skin, compared to control, demonstrating the adaptation of the muscle to the ischemia. These results would suggest that our murine hindlimb model is a reliable tool for investigating ischemia-reperfusion to improve free flap healing and survival.

### Conclusion

We have demonstrated that our Laser-Doppler Flowmetry assisted murine model can reproducibly create reversible, complete, acute hindlimb IRI. Thus, depending on the relevant clinical considerations, various ischemia levels and time periods can be simulated in experiments on mice that are easily performed and inexpensive. While it is accepted that 4 hours of ischemia time is considerably more than the accepted norm in any clinical free flap harvest, our preclinical murine model is still likely to aid in the understanding of cellular and molecular tissue alterations by IRI, and may allow for development of relevant translational protocols to improve tissue regeneration after a combined microsurgical free flap procedure.

## References

[1] Holzle F, Wolff KD, Mohr C (2008) Reconstructive oral and maxillofacial surgery. Dtsch Arztebl Int 105: 815–822. 19578412 10.3238/arztebl.2008.0815 PMC2697011

[2] Bui DT, Cordeiro PG, Hu QY, Disa JJ, Pusic A, et al (2007) Free flap reexploration: indications, treatment, and outcomes in 1193 free flaps. Plast Reconstr Surg 119: 2092–2100. 17519706 10.1097/01.prs.0000260598.24376.e1

[3] Yassin MM, Harkin DW, Barros D’Sa AA, Halliday MI, Rowlands BJ (2002) Lower limb ischemia-reperfusion injury triggers a systemic inflammatory response and multiple organ dysfunction. World J Surg 26: 115–121. 11898044 10.1007/s00268-001-0169-2

[4] Koletsis E, Chatzimichalis A, Apostolakis E, Kokkinis K, Fotopoulos V, et al (2006) In situ cooling in a lung hilar clamping model of ischemia-reperfusion injury. Exp Biol Med (Maywood) 231: 1410–1420. 16946410 10.1177/153537020623100815

[5] Pipinos, II, Judge AR, Selsby JT, Zhu Z, Swanson SA, et al (2008) The myopathy of peripheral arterial occlusive disease: Part 2. Oxidative stress, neuropathy, and shift in muscle fiber type. Vasc Endovascular Surg 42: 101–112. 18390972 10.1177/1538574408315995 PMC12282609

[6] Carroll WR, Esclamado RM (2000) Ischemia/reperfusion injury in microvascular surgery. Head Neck 22: 700–713. 11002326 10.1002/1097-0347(200010)22:7<700::aid-hed10>3.0.co;2-h

[7] Honda HM, Korge P, Weiss JN (2005) Mitochondria and ischemia/reperfusion injury. Ann N Y Acad Sci 1047: 248–258. 16093501 10.1196/annals.1341.022

[8] Hatoko M, Tanaka A, Kuwahara M, Yurugi S, Iioka H, et al (2002) Difference of molecular response to ischemia-reperfusion of rat skeletal muscle as a function of ischemic time: study of the expression of p53, p21(WAF-1), Bax protein, and apoptosis. Ann Plast Surg 48: 68–74. 11773733 10.1097/00000637-200201000-00010

[9] Crawford RS, Hashmi FF, Jones JE, Albadawi H, McCormack M, et al (2007) A novel model of acute murine hindlimb ischemia. Am J Physiol Heart Circ Physiol 292: H830–837. 17012358 10.1152/ajpheart.00581.2006

[10] Bonheur JA, Albadawi H, Patton GM, Watkins MT (2004) A noninvasive murine model of hind limb ischemia-reperfusion injury. J Surg Res 116: 55–63. 14732349 10.1016/s0022-4804(03)00232-4

[11] Mukundan C, Gurish MF, Austen KF, Hechtman HB, Friend DS (2001) Mast cell mediation of muscle and pulmonary injury following hindlimb ischemia-reperfusion. J Histochem Cytochem 49: 1055–1056. 11457933 10.1177/002215540104900813

[12] Yassin MM, Barros D’Sa AA, Parks G, Abdulkadir AS, Halliday I, et al.. (1996) Mortality following lower limb ischemia-reperfusion: a systemic inflammatory response? World J Surg 20: 961–966; discussion 966–967. 10.1007/s002689900144 8798348

[13] Wakai A, Winter DC, Street JT, O’Sullivan RG, Wang JH, et al (2001) Inosine attenuates tourniquet-induced skeletal muscle reperfusion injury. J Surg Res 99: 311–315. 11469903 10.1006/jsre.2001.6192

[14] Vignaud A, Hourde C, Medja F, Agbulut O, Butler-Browne G, et al (2010) Impaired skeletal muscle repair after ischemia-reperfusion injury in mice. J Biomed Biotechnol 2010: 724914. 20467471 10.1155/2010/724914 PMC2866363

[15] Rosenthal SM (1943) Experimental chemotherapy of burns and shock. Public Health Rep 58: 1429–1436.19315940

[16] Kyriakides C, Austen WG Jr, Wang Y, Favuzza J, Moore FD Jr, et al (2000) Neutrophil mediated remote organ injury after lower torso ischemia and reperfusion is selectin and complement dependent. J Trauma 48: 32–38. 10647562 10.1097/00005373-200001000-00006

[17] Kyriakides C, Austen W Jr, Wang Y, Favuzza J, Kobzik L, et al (1999) Skeletal muscle reperfusion injury is mediated by neutrophils and the complement membrane attack complex. Am J Physiol 277: C1263–1268. 10600778 10.1152/ajpcell.1999.277.6.C1263

[18] Kuntscher MV, Schirmbeck EU, Menke H, Klar E, Gebhard MM, et al (2002) Ischemic preconditioning by brief extremity ischemia before flap ischemia in a rat model. Plast Reconstr Surg 109: 2398–2404. 12045567 10.1097/00006534-200206000-00034

[19] Brenes RA, Jadlowiec CC, Bear M, Hashim P, Protack CD, et al.. (2012) Toward a mouse model of hind limb ischemia to test therapeutic angiogenesis. J Vasc Surg. 10.1016/j.jvs.2012.04.067 PMC3508332 22836102

[20] Kinnaird T, Stabile E, Burnett MS, Shou M, Lee CW, et al (2004) Local delivery of marrow-derived stromal cells augments collateral perfusion through paracrine mechanisms. Circulation 109: 1543–1549. 15023891 10.1161/01.CIR.0000124062.31102.57

[21] Pedowitz RA (1991) Tourniquet-induced neuromuscular injury. A recent review of rabbit and clinical experiments. Acta Orthop Scand Suppl 245: 1–33. 1950503

